# Diurnal rhythm of blood pressure among Nigerians with hypertension using 24-hour ambulatory blood pressure monitoring

**DOI:** 10.11604/pamj.2020.36.240.24088

**Published:** 2020-08-04

**Authors:** Ifeoluwa Amjo, Rasaaq Ayodele Adebayo, Olumide Akinniyi Akinyele, Oladiipo Ayoola Olanipekun, Obafemi Sunday Adesanya, Oyeronke Titilope Williams, Suraj Adefabi Ogunyemi, Anthony Olubunmi Akintomide, Olufemi Eyitayo Ajayi, Michael Olabode Balogun, Busayo Onafowoke Oguntola, Ikponmwosa Godfrey Akhionbare, Lukman Obasanjo Adebiyi

**Affiliations:** 1 Cardiology Unit, Department of Medicine, Obafemi Awolowo University Teaching Hospitals Complex (OAUTHC), Ile-Ife, Osun State, Nigeria

**Keywords:** Dipping pattern, nocturnal hypertension, awake blood pressure, asleep blood pressure, 24-h ambulatory blood pressure monitoring, Nigeria

## Abstract

**Introduction:**

hypertension is the most common cardiac disease in Nigeria. There are very limited studies in Nigeria on the use of 24-hour ambulatory blood pressure monitoring (24-h ABPM) for evaluation of hypertensive patients. Twenty four-hour ABPM, unlike office blood pressure (OBP), can assess diurnal variation using parameters like awake blood pressure (BP), asleep (nocturnal) BP, mean 24-hour BP and dipping pattern. This can help in assessment of increased cardiovascular risk and management of hypertensive patients. We purposed to assess the diurnal rhythm of BP among Nigerians with hypertension.

**Methods:**

this was a prospective cross-sectional study. Consecutive 77 hypertensive subjects were studied using Schiller MT-300 for 24-h ABPM.

**Results:**

out of the 77 patients reviewed, 39 (50.6%) were females. The mean age was 50.9 years (SD 13.5). The mean awake systolic and diastolic BP were 135.6mmHg (SD 15.0) and 83.2mmHg (SD 10.0) respectively; mean asleep systolic and diastolic BP were 127.6mmHg (SD 17.9) and 76.2mmHg (SD 12.2) respectively; and mean 24-h systolic and diastolic BP were 133.6mmHg (SD 15.3) and 81.4mmHg (SD 10.2) respectively. Awake BP was elevated in 59.7% of study subjects. Elevated awake systolic BP and awake diastolic BP were present in 50.6% and 41.6% of the study population. Nocturnal (asleep) BP was elevated in 79.2%. Non-dipping pattern was the most prevalent pattern at 55.8%, followed by dipping (24.7%), reverse dipping (15.6%) and extreme dipping (3.9%).

**Conclusion:**

a high proportion had nocturnal hypertension (79.2%) and non-dipping pattern was the most prevalent pattern (55.8%). Mean awake systolic BP, mean asleep systolic and diastolic BP and mean 24-h systolic and diastolic BP were elevated. The use of 24-h ABPM will enhance assessment of increased cardiovascular risk and management of Nigerians with hypertension.

## Introduction

Hypertension is the most common cardiac disease in Nigeria [1,2]. It is the commonest risk factor for stroke, heart failure and chronic kidney disease in Nigeria [3]. Evaluation of hypertensive patients is commonly done using office BP, especially in resource poor settings like in our environment. Twenty four-hour ABPM has been reported to predict cardiovascular events and is superior to office BP in predicting cardiovascular mortality and events in hypertensive patients [4-7]. There is no record of the pattern of diurnal variation in BP of hypertensives in our hospital. In addition, there are limited studies in Nigeria and the West Africa sub-region on the use of 24-h ABPM in evaluation of hypertensive patients. We therefore studied the diurnal rhythm of BP among hypertensive patients referred for 24-h ABPM at Cardiac Care Unit (CCU) ABPM Laboratory of Obafemi Awolowo University Teaching Hospitals Complex (OAUTHC), Ile-Ife, Osun State, Nigeria to determine the pattern of diurnal variations in BP of these patients in our centre. We believe the findings will further encourage the use of 24h-ABPM in evaluating African patients with hypertension, so as to improve on the assessment of their increased cardiovascular risk and also blood pressure management. This information obtained will also add to the national, regional and global database on 24-h ABPM from an African perspective.

## Methods

**Design and study population:** this is a prospective, cross-sectional study involving 77 hypertensive subjects aged 18 years and above referred for 24-h ABPM at the cardiac care unit of OAUTHC. Subjects were recruited consecutively. Each subject had office (clinic) blood pressure (BP) measured by the auscultatory method in both arms using table top aneroid sphygmomanometer while the patient was relaxed and sitting. The arm with the higher reading was taken as the patients BP after an average of three readings. Systolic and diastolic (phase V) BP were determined to the nearest 2mmHg. This was followed by 24-h ABPM using Schiller MT-300 according to the recommendations of European society of hypertension practice guidelines for ambulatory blood pressure monitoring [8]. The procedure and handling of the ABPM machine was explained to the subjects after which patients´ details were entered and the monitor initialized. The machine was programmed to read half hourly from 7 a.m. to 10 p.m. and hourly from 10 p.m. to 7 a.m. An appropriate cuff size was chosen and this was applied immediately to the subject´s non-dominant bare arm. Subjects were discharged home to continue their normal activities and return after 24 hours for retrieval of the Holter monitor. They were advised to remain still during measurements and to abstain from smoking, alcohol and consumption of caffeinated drinks throughout the period of study. The device was removed after 24-hours and data downloaded into the computer. A minimum of 70% usable BP recordings are required for a valid ABPM measurement. Hypertension was defined as office blood pressure ≥140mmHg (systolic) and/or ≥90mmHg (diastolic); mean 24-hour blood pressure ≥130mmHg (systolic) and/or ≥80mmHg (diastolic); mean awake blood pressure ≥135mmHg (systolic) and/or ≥85mmHg (diastolic); and mean asleep (nocturnal) blood pressure ≥120mmHg (systolic) and/or ≥70mmHg (diastolic). Dipping pattern was categorized into dipping (>10% reduction in night SBP compared to awake SBP), non-dipping (<10%), reverse dipping (<0%) and extreme dipping (>20%) [8,9].

**Data analysis:** the data was analysed using STATA statistical software package version 14. Data were represented using descriptive statistics such as table, bar chart and pie chart. Categorical variables were expressed as proportions and percentages while continuous variables were expressed as means with standard deviations (SD).

**Ethics:** ethical approval was obtained from ethics and research committee of OAUTHC with approval protocol number ERC/2017/06/07. All participants gave informed written consent.

## Results

**Figure 1:**
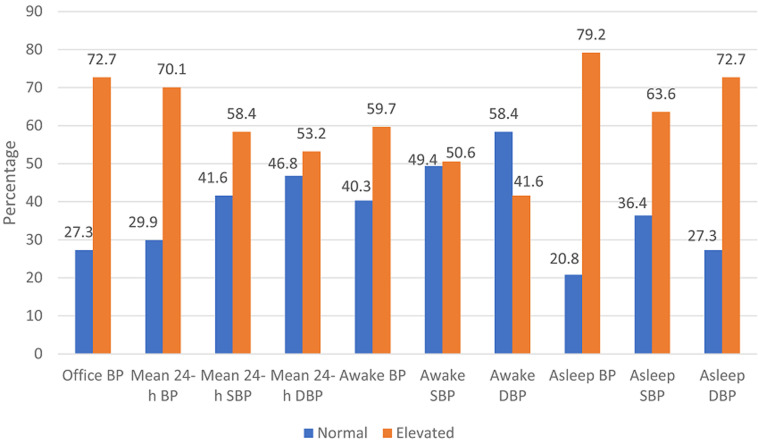
24-hour ambulatory blood pressure and office blood pressure parameters in study subject

**Figure 2:**
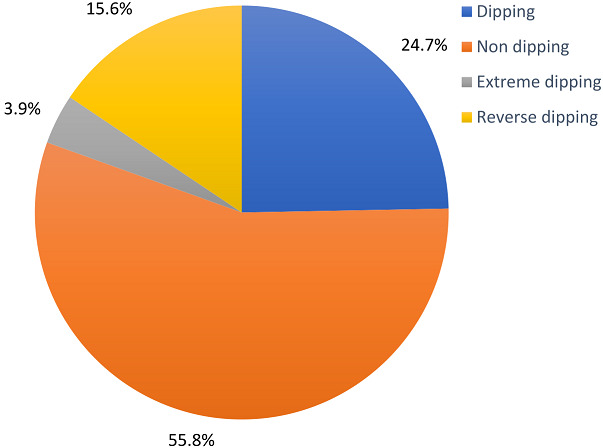
dipping pattern in study participants using systolic blood pressure

A total of 77 participants were recruited for the study which included 39 (50.6%) female and 38 (49.4%) male hypertensive subjects with the mean age of 50.9 years (SD 13.5) and mean body mass index (BMI) of 28.6kg/m^2^ (SD 4.7) (Table 1). Common indications for 24-h ABPM referral to our CCU include: diagnosis of hypertension; diagnosis of white coat hypertension; assessment of treatment of hypertension. The mean awake systolic and diastolic BP were 135.6mmHg (SD 15.0) and 83.2mmHg (SD 10.0) respectively; mean asleep systolic and diastolic BP were 127.6mmHg (SD 17.9) and 76.2mmHg (SD 12.2) respectively; and mean 24-h systolic and diastolic BP were 133.6mmHg (SD 15.3) and 81.4mmHg (SD 10.2) respectively (Table 1). Awake blood pressure was elevated (daytime hypertension) in 59.7% of study subjects while 50.6% and 41.6% had elevated awake systolic and diastolic blood pressure respectively (Figure 1). Asleep blood pressure was elevated (nocturnal hypertension) in 79.2% of study subjects while 63.6% and 72.7% had elevated asleep systolic and diastolic blood pressure respectively (Figure 1). Dipping, non-dipping, extreme dipping and reverse dipping constituted 24.7%, 55.8%, 3.9% and 15.6% respectively (Figure 2).

**Table 1:** baseline characteristics of the study population

PARAMETERS	FREQUENCY(n=77)
Female n (%)	39 (50.6%)
Age (years)	50.9 (SD 13.5)
BMI (Kg/m^2^)	28.6 (SD 4.7)
Weight (Kg)	79.3 (SD 14.6)
Height (m)	1.7 (SD 0.1)
**Office BP (mmHg)**	
Systolic	147.2 (SD 22.5)
Diastolic	88.9 (SD 14.6)
**Mean 24-hour BP (mmHg)**	
Systolic	133.6 (SD 15.3)
Diastolic	81.4 (SD 10.2)
**Awake BP**	
Systolic	135.6 (SD 15.0)
Diastolic	83.2 (SD 10.0)
**Asleep BP (mmHg)**	
Systolic	127.6 (SD 17.9)
Diastolic	76.2 (SD 12.2)

SD= Standard deviation, BMI= Body mass index, BP= Blood pressure

## Discussion

This was a cross-sectional study of 77 hypertensive subjects. The mean age of the study population was 50.9 years (SD 13.5). This was similar to the mean age reported by Ajayi *et al*. [10] and Isiguzo *et al*. [11] in Nigeria. The mean BMI was 28.6kg/m^2^ (SD 4.7). Majority (70.1%) of the study subjects had elevated mean 24-hour ambulatory blood pressure. A similarly high proportion of 76.6% was observed by Isiguzo *et al*. [11] and 58% by Adeoye *et al*. [12], both in Nigeria. Yang *et al*. [13] in a population-based cohort study of 11,135 adults from Europe, Asia and South America reported that higher mean 24-h ABPM measurement was significantly associated with greater risks of death and composite cardiovascular outcome (non-fatal coronary event, heart failure and stroke). This suggests a lot of our subjects may have increased cardiovascular risk. A high proportion of study participants (50.6%) had elevated awake systolic BP compared with 41.6% who had elevated awake diastolic BP, meaning elevated systolic BP in the daytime was more common compared with diastolic BP. Elevated awake systolic hypertension has been reported to be associated with cardiovascular mortality. Furthermore, it is also an important risk factor for stroke and other cardiovascular events among African Americans [5,14]. Nocturnal hypertension was common among our study subjects and present in 79.2% of study subjects. This finding was at variance with a prevalence of 22.7% reported by Isiguzo *et al*. [11]. A significant association has been established between the presence of nocturnal hypertension and cardiovascular mortality [5].

A higher night time (nocturnal) BP measurement was reported to be significantly associated with greater risks of death and composite cardiovascular outcomes (non-fatal coronary event, heart failure and stroke) in a large population-based cohort study involving adults in Europe, Asia and South America [13]. Asleep (nocturnal) diastolic BP was elevated in 72.7% compared with 63.6% with elevated asleep (nocturnal) systolic BP, though both were still largely elevated in the study participants. Nighttime (nocturnal) ABPM provides additional predictive information over daytime ABPM [5]. The nighttime systolic BP is the most potent predictor of cardiovascular event and mortality [15]. Among African Americans higher nighttime (nocturnal/asleep) systolic BP was associated with all-cause mortality, while higher night time (nocturnal/asleep) systolic and diastolic BP were associated with increased risk of cardiovascular disease (coronary heart disease and stroke) [14]. This further reinforces the importance of evaluating hypertensives with 24-h ABPM. In this study, non-dipping pattern (55.8%) was the most prevalent pattern, followed by dipping (24.7%) and reverse dipping (15.6%), while extreme dipping was the least prevalent (3.9%). The same sequence was reported by Isiguzo *et al*. [11] with 47.8%, 30.4%, 15.2% and 6.5% respectively. A non-dipping pattern of BP is associated with significantly higher frequency of stroke than dipping [16].

It also has more hypertension-induced organ damage such as left ventricular hypertrophy, microalbuminuria and decreased arterial compliance compared with dipping [17]. Reverse dipping constituted 15.6% in this study. A similar prevalence of 12% was reported by Fagard *et al*. [18] in Europe. Nocturnal increase in sympathetic nervous system activity has been mentioned as one of the mechanisms responsible for the development of reverse dipping pattern [19]. Fagard *et al*. [18] in a meta-analysis of four studies on hypertensives in Europe reported that all-cause mortality was lower in extreme dipping than in normal dipping pattern. In addition, reverse dippers are at a higher risk of cardiovascular disease than dippers, independently of 24-hour ambulatory blood pressure and other confounding factors. In 362 hypertensive patients studied by Yan *et al*. [20], reverse dipping pattern was found to be directly associated with lacunar infarction. Results from the MAPEC study and the hygia chronotherapy trial in hypertensives have shown that bedtime use of ≥1 BP-lowering medications, compared to conventional upon awaking use of medications in hypertensives, more effectively improved BP control, better decreased the prevalence of non-dipping, elevated asleep BP (nocturnal hypertension) and most importantly, it significantly reduced cardiovascular morbidity (heart failure, myocardial infarction and stroke) and mortality [21,22]. This is an option that may help in achieving better diurnal rhythm among hypertensives.

## Conclusion

A high proportion had nocturnal hypertension (79.2%) and non-dipping pattern was the most prevalent pattern (55.8%). Mean awake systolic BP, mean asleep systolic and diastolic BP and mean 24-h systolic and diastolic BP were elevated. The use of 24-h ABPM will enhance assessment of increased cardiovascular risk and management of Nigerians with hypertension.

### What is known about this topic

- The presence of left ventricular hypertrophy in hypertensive Nigerians was associated with higher mean ambulatory systolic BP;
- A stronger relationship exists between 24-hour mean blood pressure and left ventricular mass in hypertensive Nigerians.

### What this study adds

- Literature on 24-h ABPM in hypertensive patients in Nigeria and sub-Saharan Africa is still unfolding and this study will contribute to the growing body of knowledge;
- In particular, this study helps in identifying the high prevalence of increased cardiovascular risk factors such as elevated mean awake systolic blood pressure, elevated mean 24-h BP, nocturnal hypertension and non-dipping pattern in Nigerians with hypertension.

## References

[1] Ojji D, Stewart S, Ajayi S, Manmak M, Sliwa K (2013). A predominance of hypertensive heart failure in the Abuja heart study cohort of urban Nigerians: a prospective clinical registry of 1515 de novo cases. Eur J Heart Fail.

[2] Adebayo RA, Akinwusi PO, Balogun MO, Akintomide AO, Adeyeye VO, Abiodun OO (2013). Two-dimensional and doppler echocardiographic evaluation of patients presenting at Obafemi Awolowo University Teaching Hospitals Complex, Ile-Ife, Nigeria: a prospective study of 2501 subjects. Int J Gen Med.

[3] Ogah OS, Arije A, Xin X, Beaney T, Adebiyi A, Sani MU (2019). May Measurement Month 2017: screening for hypertension in Nigeria sub-Saharan Africa. Eur Hear J Suppl.

[4] Clement DL, De Buyzere ML, De Bacquer DA, De Leeuw PW, Duprez DA, Fagard RH (2003). Prognostic value of ambulatory blood-pressure recordings in patients with treated hypertension. N Engl J Med.

[5] Dolan E, Stanton A, Thijs L, Hinedi K, Atkins N, McClory S (2005). Superiority of ambulatory over clinic blood pressure measurement in predicting mortality: the Dublin outcome study. Hypertension.

[6] Banegas JR, Ruilope LM, De La Sierra A, Vinyoles E, Gorostidi M, De La Cruz JJ (2018). Relationship between clinic and ambulatory blood-pressure measurements and mortality. N Engl J Med.

[7] Mancia G, Verdecchia P (2015). Clinical value of ambulatory blood pressure: evidence and limits. Circ Res.

[8] Parati G, Stergiou G, O´Brien E, Asmar R, Beilin L, Bilo G (2014). European society of hypertension practice guidelines for ambulatory blood pressure monitoring. J Hypertens.

[9] Williams B, Mancia G, Spiering W, Rosei EA, Azizi M, Burnier M (2018). 2018 ESC/ESH guidelines for the management of arterial hypertension. Eur Heart J.

[10] Ajayi OE, Ajayi EA, Akintomide OA, Adebayo RA, Ogunyemi SA, Oyedeji AT (2011). Ambulatory blood pressure profile and left ventricular geometry in Nigerian hypertensives. J Cardiovasc Dis Res.

[11] Isiguzo G, Baugh D, Nwuruku G, Mezue K, Madu C, Madu E (2016). Initial experience with 24-h ambulatory blood pressure monitoring in Nigerian patients with hypertension. Niger J Cardiol.

[12] Adeoye AM, Tayo BO, Owolabi MO, Adebiyi AA, Lackland DT, Cooper R (2018). Ambulatory blood pressure threshold for black Africans: more questions than answers. J Clin Hypertens.

[13] Yang W-Y, Melgarejo JD, Thijs L, Zhang Z-Y, Boggia J, Wei F-F (2019). Association of office and ambulatory blood pressure with mortality and cardiovascular outcomes. JAMA.

[14] Yano Y, Tanner RM, Sakhuja S, Jaeger BC, Booth JN, Abdalla M (2019). Association of daytime and nighttime blood pressure with cardiovascular disease events among African American individuals. JAMA Cardiol.

[15] De La Sierra A, Banegas JR, Segura J, Gorostidi M, Ruilope LM, CARDIORISC Event Investigators (2012). Ambulatory blood pressure monitoring and development of cardiovascular events in high-risk patients included in the Spanish ABPM registry: the cardiorisc event study. J Hypertens.

[16] O´Brien E, Sheridan J, O´Malley K (1988). Dippers and non-dippers. Lancet.

[17] Redon J, Lurbe E (2008). Nocturnal blood pressure versus nondipping pattern: what do they mean. Hypertension.

[18] Fagard RH, Thijs L, Staessen JA, Clement DL, De Buyzere ML, De Bacquer DA (2009). Night-day blood pressure ratio and dipping pattern as predictors of death and cardiovascular events in hypertension. J Hum Hypertens.

[19] Cuspidi C, Sala C, Tadic M, Gherbesi E, De Giorgi A, Grassi G (2017). Clinical and prognostic significance of a reverse dipping pattern on ambulatory monitoring: an updated review. J Clin Hypertens.

[20] Yan B, Peng L, Dong Q, Zheng F, Yang P, Sun L (2015). Reverse-dipper pattern of blood pressure may predict lacunar infarction in patients with essential hypertension. Eur J Neurol.

[21] Hermida RC, Ayala DE, Mojón A, Fernández JR (2010). Influence of circadian time of hypertension treatment on cardiovascular risk: Results of the MAPEC study. Chronobiol Int.

[22] Hermida RC, Crespo JJ, Domínguez-Sardiña M, Otero A, Moyá A, Ríos MT (2019). Bedtime hypertension treatment improves cardiovascular risk reduction: the Hygia chronotherapy trial. Eur Heart J.

